# Sheep β-Defensin 2 Regulates *Escherichia coli* F17 Resistance via NF-κB and MAPK Signaling Pathways in Ovine Intestinal Epithelial Cells

**DOI:** 10.3390/biology10121356

**Published:** 2021-12-20

**Authors:** Ling Ge, Shuangxia Zou, Zehu Yuan, Weihao Chen, Shanhe Wang, Xiukai Cao, Xiaoyang Lv, Tesfaye Getachew, Joram M. Mwacharo, Aynalem Haile, Wei Sun

**Affiliations:** 1 College of Animal Science and Technology, Yangzhou University, Yangzhou 225000, China; gl1024winnie@163.com (L.G.); zshx201422@163.com (S.Z.); 18552133709@163.com (W.C.); shanhe12315@163.com (S.W.); 2 Joint International Research Laboratory of Agriculture and Agri-Product Safety of Ministry of Education, Yangzhou University, Yangzhou 225000, China; yuanzehu1988@163.com (Z.Y.); cxkai0909@163.com (X.C.); dx120170085@yzu.edu.cn (X.L.); 3 International Centre for Agricultural Research in the Dry Areas, Addis Ababa 999047, Ethiopia; t.getachew@cgiar.org (T.G.); j.mwacharo@cgiar.org (J.M.M.); a.haile@cgiar.org (A.H.)

**Keywords:** sheep, *SBD*-2, *Escherichia coli* F17, inflammation, NF-κB pathway, MAPK pathway

## Abstract

**Simple Summary:**

This study was conducted to explore the antibacterial ability of sheep β-defensin 2 (*SBD*-2) against *E. coli* F17 infection in ovine intestinal epithelial cells (OIECs). Our data revealed that *E. coli* F17 induces *SBD*-2 expression in OIECs in vitro, which appears to be mediated through the activation of the signaling pathways NF-κB and MAPK. Our results provide a novel insight for the functionality of *SBD*-2, which could be useful for developing anti-infective drugs and/or breeding for *E. coli* diarrhea disease-resistant sheep.

**Abstract:**

*Escherichia coli* (*E. coli*) F17 is a member of enterotoxigenic *Escherichia coli*, which can cause massive diarrhea and high mortality in newborn lambs. β-defensin is mainly produced by the epithelial tissue of the gastrointestinal tract in response to microbial infection. However, the molecular mechanism of sheep β-defensin 2 (*SBD*-2) against *E. coli* F17 remains unclear. This study aims to reveal the antibacterial ability of *SBD*-2 against *E. coli* F17 infection in sheep. Firstly, we established the culture system of ovine intestinal epithelial cells (OIECs) in vitro, treated with different concentrations of *E. coli* F17 for an indicated time. Secondly, we performed RNA interference and overexpression to investigate the effect of *SBD*-2 expression on *E. coli* F17 adhesion to OIECs. Finally, inhibitors of NF-κB and MAPK pathways were pre-treated to explore the possible relationship involving in *E. coli* F17 infection regulating *SBD*-2 expression. The results showed that *E. coli* F17 markedly (*p* < 0.01) upregulated the expression levels of SBD-2 mRNA and protein in a concentration- and time-dependent manner. Overexpression of *SBD*-2 contributed to enhancing *E. coli* F17 resistance in OIECs, while silencing *SBD*-2 dramatically improved the adhesion of *E. coli* F17 to OIECs (*p* < 0.05 or *p* < 0.01). Furthermore, *E. coli* F17 stimulated *SBD*-2 expression was obviously decreased by pre-treatment with NF-κB inhibitor PDTC, p38 MAPK inhibitor SB202190 and ERK1/2 MAPK inhibitor PD98095 (*p* < 0.05 or *p* < 0.01). Interestingly, adhesion of *E. coli* F17 to OIECs were highly enhanced by pre-treated with PDTC, SB202190 and PD98095. Our data suggested that *SBD*-2 could inhibit *E. coli* F17 infection in OIECs, possibly through NF-κB and MAPK signaling pathways. Our results provide useful theoretical basis on developing anti-infective drug and breeding for *E. coli* diarrhea disease-resistant sheep.

## 1. Introduction

Enterotoxigenic *Escherichia coli* (ETEC) F17 is one of the most common pathogens causing *E. coli* diarrhea in lambs. Its fatality rate is very high, which seriously hinders the production and profit of large-scale sheep farms [1,2]. ETEC is characterized by its ability to produce two types of virulence factor: adhesin and enterotoxin [3]. Once colonized by adhesin in the intestines of neonatal animals, ETEC will produce two major classes of enterotoxins, namely, heat-labile toxin (LT) and heat-stable toxin (ST). According to their antigenic properties, the main adhesins can be classified into different sub-types, such as F4 (also designated K88), F5 (K99), F6 (987p), F17 (Fy), F18 and F41 [4]. F17 fimbriae were first found in diarrhea calves, which are typically colonized in small intestinal mucosa to produce STa enterotoxin, leading to diarrhea in lambs [5].

Defensins, as the first line of defense against pathogens, have broad-spectrum bactericidal effect, especially in killing Gram-negative and Gram-positive bacteria [6]. Due to the side effects of traditional antibiotics and the emergence of drug-resistant strains [7], the use of defensin as a new drug or functional target gene to breed disease-resistant animals may be helpful to control bacterial infection. β-defensins are mainly distributed in the epithelial tissues of the gastrointestinal tract, which had been found in human, pigs, cattle, sheep, etc. [8]. In sheep, two types of β-defensins, sheep β-defensin 1 (*SBD*-1) and sheep β-defensin 2 (*SBD*-2), have been described [9]. However, the function of *SBD*-2 involved with *E. coli* F17 infection in sheep has not been reported. In other animals, accumulated evidence showed that β-defensins not only have strong antibacterial activity, but also participate in regulating inflammatory response. It has been previously reported that human β-defensin 118 could reduce the inflammation and intestinal injury in mice induced by *E.coli* F4 [10]. For the inflammatory response of *E. coli*, it is mainly induced by lipopolysaccharide (LPS), which can be recognized by Toll-like receptors (TLRs) family, mainly resulting in the activation of nuclear factor-kappa B (NF-κB) and mitogen-activated protein kinases (MAPK) signaling [11]. In a study related to *HBD-3*, the researchers found that *HBD-3* could down-regulate the expression of cytokines in macrophages through inhibiting the activation of NF-κB and MAPK pathways induced by LPS [12]. Another report revealed that *PBD-2* could improve inflammatory response by affecting the activation of NF-κB signal pathway [13]. Based on the above research progress, we hypothesized that *SBD*-2 could play a potential role in regulating *E. coli* F17 infection in sheep and two inflammatory pathways were involved in the process of *E. coli* F17 infection.

Thus, in this study, we constructed the model of ovine intestinal epithelial cells (OIECs) infected by *E. coli* F17, and verified the relationship between the expression of *SBD*-2 and *E. coli* F17 infection in sheep using RT-PCR and ELISA analysis. Furthermore, we performed RNA interference and overexpression to inquiry the effect of *SBD*-2 expression on *E. coli* F17 adhesion to OIECs. Further, we used RT-PCR analysis to detect whether NF-κB and MAPK pathways were activated after *E. coli* F17 stimulation. Then, we conducted pathway inhibition experiment to study the effect on *SBD*-2 expression. Finally, colony count analysis was conducted to explore the influence of pathway inhibition on the adhesion of *E. coli* F17 to OIECs. This study not only comprehensively explored the essential role of *SBD*-2 regulating *E. coli* F17 infection in OIECs, but also deeply studied the status of the NF-κB and MAPK pathways in this process. Our results could provide feasible treatment direction for *E. coli* F17 resistance in lambs and theoretical basis on breeding for *E. coli* diarrhea disease-resistant sheep.

## 2. Materials and Methods

### 2.1. Experimental Sample and Ethical Statement

OIECs used in this experiment were isolated from two healthy lambs of Hu sheep at the age of 3 to 5 days (Jiangsu Xilaiyuan Ecological Agriculture Co., Ltd., Taizhou, China). The detailed isolation method was documented in Figure S1. Purification and identification work had been finished in the laboratory of our research group. *E. coli* F17 strain (DN1502) was offered by Prof. Dr. Dongfang Shi, Northeast Agricultural University (Harbin, China).

The Institutional Animal Care and Use Committee (IACUC) of the government of Jiangsu Province (Permit Number 45) and the Ministry of Agriculture of China (Permit Number 39) approved the animal study proposal. All experimental procedures were conducted in strict compliance with the recommendations of the Guide for the Care and Use of Laboratory Animals of Jiangsu Province and of the Animal Care and Use Committee of the Chinese Ministry of Agriculture.

### 2.2. Cell Culture and E. coli F17 Stimulation in OIECs

OIECs were cultured with a DMEM/F12 medium supplemented with 10% fetal bovine serum and 1% penicillin-streptomycin (All from Sigma-Aldrich, St. Louis, MO, USA) in 5% CO_2_ atmosphere at 37 °C. The growth of cells was observed under an inverted fluorescence microscope. *E. coli* F17 strain, which was preserved with glycerin, was inoculated into a Luria-Bertani (LB) agar Petri dish and incubated for 16–18 h at 37 °C. Then, a single colony was picked up with the end of a sterilization inoculation ring and inoculated it into LB liquid medium, and then they were incubated overnight on a shaker with 150 rpm (more details about *E. coli* F17 were shown in Table S1).

In order to determine the best infection condition related to *SBD*-2 gene, here we designed different infection concentrations and times. Cells were inoculated to 6-well plates, cultured in DMEM/F12 medium without serum and antibiotics, and then performed *E. coli* F17 stimulation. Firstly, OIECs were treated with five different concentrations of *E. coli* F17, namely, 0 CFU/mL, 10^5^ CFU/mL, 10^6^ CFU/mL, 10^7^ CFU/mL and 10^8^ CFU/mL, respectively. Then, based on the best concentration obtained from the previous experiment, OIECs were treated with six different infection times, namely, 0 h, 2 h, 4 h, 6 h, 8 h and 10 h. Cells collection was used for RT-PCR analysis and Enzyme-linked Immunosorbent Assay (ELISA) to detect mRNA and protein expression levels of SBD-2, respectively. Specific primers for RT-PCR were shown in Table S2.

### 2.3. Plasmid Construction and RNA Oligonucleotides

According to the coding sequence (CDS) of *SBD*-2 in NCBI (https://www.ncbi.nlm.nih.gov/ (accessed on 23 November 2021)), primers were designed by premier primer 5.0 software (Premier Biosoft International, Palo Alto, CA, USA) and were documented in Table S3. Full-length DNA encoding *SBD*-2 were amplified using PrimeSTAR Max DNA Polymerase reagent (Takara, Kusatsu, Shiga, Japan). The obtained PCR product was purified and recovered by SanPrep column PCR product purification kit (Sangon Biotech, Shanghai, China) and then was sequenced by Beijing Tsingke Biotechnology Co., Ltd. (Nanjing, China). The pGH plasmid (Generay, Shanghai, China) and *SBD*-2 target fragment were digested with restriction enzyme QuickCut *Eco*R V (Takara), then connected the target fragment into the linear vector according to the instruction of DNA Ligation Kit Ver.2.1 kit (Takara). After enzyme digestion identification and sequencing verification, the successfully constructed cloning plasmid was named pGH-SBD-2. A schematic diagram for the construction of *SBD*-2 overexpression vector was shown in Figure S2. Small-interfering ribonucleic acids (siRNAs) of *SBD*-2 together with negative control (NC) were synthesized by GenePharma Pharmaceutical Technology Co., Ltd. (Shanghai, China). All sequences were shown in Table S4.

### 2.4. Cell Transfection

When the cell confluence reached 50–60%, pGH-SBD-2 plasmid and siRNAs of *SBD*-2 were transfected into cells with jetPRIME transfection reagent (Polyplus transfection, Illkirch, France) following manufacturer’s instructions. Each transfection had been treated with at least three replications. After 24 h, cells were collected for RT-PCR analysis to verify transfection efficiency.

### 2.5. Pathway Inhibitors Blocking Signaling Pathway Test

To investigate the function of NF-κB and MAPK signaling pathway in the process of *SBD*-2 regulating *E. coli* F17 infection in OIECs, pathway inhibitors (Beyotime, Shanghai, China) were added into four groups of well-growing OIECs, namely, blank control group, positive control group, negative control group and experimental group. The detailed treatment of each group was shown in Table 1. Then, cells were collected to quantify the mRNA expression levels of *p65*, *p50*, *p38*, *ERK1* and *JNK*. Using these cells, the mRNA and protein expression levels of SBD-2 were been quantified as well. The primers used for RT-PCR were documented in Table S2.

### 2.6. Total RNA Extraction and Real-Time PCR (RT-PCR)

Total RNA was extracted from cells using the TRIzol reagent (TIANGEN, Beijing, China). The purity and concentration were detected by 1% agarose gel electrophoresis and NanoReady spectrophotometer (Life Real, Hangzhou, China). All RNA samples were stored at −80 °C. Reverse transcription was implemented using the FastKing gDNA Dispelling RT Super Mix (TIANGEN). The reverse transcription reaction system and reaction condition were summed up in Table S5. RT-PCR was conducted using the 2× TSINGKE Master qPCR Mix (SYBR Green I) (TSE201, Tsingke, Beijing, China). The detailed amplification program was shown in Table S5. The *GAPDH* was used as an internal reference gene. RT-PCR was implemented by using CFX96 Connect™ Real-Time System (BIO-RAD, CA, USA). All RT-PCR results were analyzed using 2^−∆∆Ct^ method [14].

### 2.7. Enzyme-Linked Immunosorbent Assay (ELISA)

To assess SBD-2 mass concentration, the cell culture medium was collected to implemented ELISA after plasmid transfection and *E. coli* F17 infection, referring to the sheep β-Defensin 2 enzyme-linked immunosorbent assay detection kit instructions (mlbio, Shanghai, China), following specific steps. Firstly, the above cell medium was centrifuged at 4 °C, 2000–3000 rpm for 20 min and supernatant was saved as samples. Secondly, 50 µL standard substance of different concentrations was added into the standard wells; meanwhile, 50 µL sample was added into the sample wells except the blank wells. Thirdly, 100 µL enzyme-labeled reagent was added into standard wells and sample wells except the blank wells, and they were incubated at 37 °C for 1 h. Then, 350 µL washing liquid was added into each well for 1 min and absorbent paper was used to pat dry, 5 times repeatedly. Finally, 50 µL substrate A and B were added into each well, and they were incubated at 37 °C for 15 min in the dark, then 50 µL stop solution was added into each well. OD value of each well was measured at 450 nm wavelength in the microplate reader at 26 °C within 15 min (Tecan, Shanghai, China). Additionally, the OD value of the measured standard substance was used as the abscissa and the concentration value was used as the ordinate to draw the standard curve.

### 2.8. Plate Counting Method for Bacteria Enumeration

To assess adhesion of *E. coli* F17 to OIECs, plate count was performed according to Jouve et al. [15] after plasmid transfection and *E. coli* F17 infection. First of all, cells were washed gently three times with PBS buffer (Sigma-Aldrich). Then, 300 µL 0.5% Triton X-100 (Solarbio, Beijing, China) were added into each well to lyse the cells for 30 min, and the cell lysate was collected. Next, 200 µL PBS buffer were added to wash twice and a total of 700 µL liquid were collected into a 1.5 mL sterile centrifuge tube, and they were mixed uniformly. Last, liquid was diluted by multiple times, then the diluent was spread on LB Petri dish, and they were inverted in a constant temperature incubator at 37 °C overnight. The number of colonies were counted.

### 2.9. Statistical Analysis

Before statistical analysis, the normality of data were been tested by Kolmogorov–Smirnov Test using SPSS 25.0. An analysis of variance (ANOVA) was applied to analyze the differences of *SBD*-2 expression level in OIECs between the *E. coli* F17 stimulation group and non-treated group with different bacteria concentration and infection times by using SPSS 25.0 software (SPSS, Inc., Chicago, IL, USA). Independent *t* test was performed to analyze the differences of *SBD*-2 expression level in OIECs between pGH group and pGH-SBD-2 group, the differences of *SBD*-2 expression level in OIECs between negative control group and siRNA-SBD-2 group, the differences of number of *E. coli* F17 colonies between pGH group and pGH-SBD-2 group, and the differences of number of *E. coli* F17 colonies between negative control group and siRNA-SBD-2 group. Independent *t* test was performed to analyze the differences of genes (*p65*, *p50*, *p38*, *ERK1/2*, *JNK*) mRNA expression levels in OIECs between non-treated group and *E. coli* F17 infection group, the differences of *p50*, *p38*, *ERK1/2* and *SBD*-2 expression levels in OIECs between non-treated group and positive control group, negative control group and experimental group, respectively. Independent *t* test was performed to analyze the differences of number of *E. coli* F17 colonies between non-treated group and pathway inhibitors treatment group. Results were represented as mean ± SD (* denotes *p* < 0.05, significant difference; ** denotes *p* < 0.01, extremely significant difference).

## 3. Results

### 3.1. Effect of E. coli F17 Different Infection Concentration and Time on SBD-2 Expression

**Figure 1:**
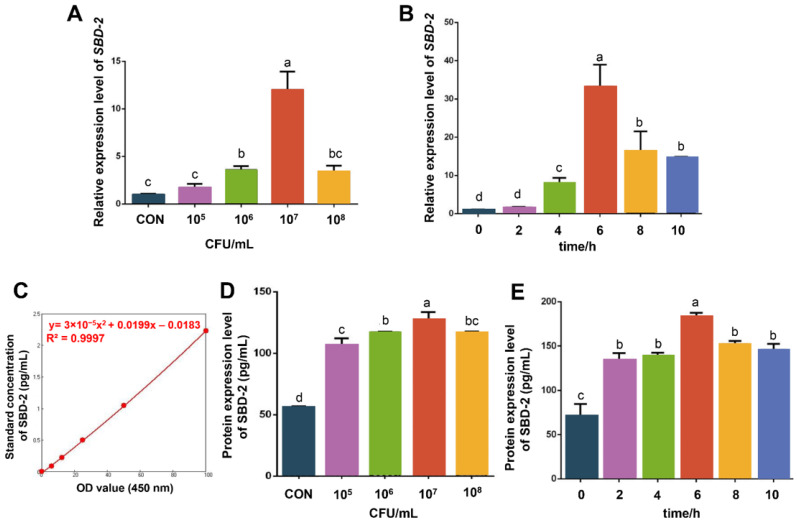
*E. coli* F17 stimulates mRNA and protein expression levels of *SBD-2.* (**A**) *SBD*-2 mRNA expression levels detected by RT-PCR in OIECs treated with the indicated concentrations of *E. coli* F17 compared with non-treated controls. (**B**) *SBD*-2 mRNA expression levels detected by RT-PCR in OIECs treated with *E. coli* F17 (10^7^ CFU/mL) for various time intervals compared with non-treated controls. (**C**) Standard curve drawn by SBD-2 protein concentration value (*y* axis) and OD value at 450 nm (*x* axis). (**D**) SBD-2 protein expression levels detected by ELISA in OIECs treated with the indicated concentrations of *E. coli* F17 compared with non-treated controls. (**E**) SBD-2 protein expression levels detected by ELISA in OIECs treated with *E. coli* F17 (10^7^ CFU/mL) for various time intervals compared with non-treated controls. Mean values with different letters in the same row are significantly different (*p* < 0.05) according to Duncan’s multiple range test; data were shown as mean ± SD, *n* = 3 biological replicates.

The resuscitated OIECs had consistent normal morphology and good activity, growing adherently and closely connected (Figure S3). They could be used in the later experiment. Using 1% agarose gel electrophoresis to check the quality of the total RNA extracted from cells, clear 28 S and 18 S bands can be observed, indicating that the integrity of the extracted RNA is of high quality (Figure S4). As clearly reflected in Figure 1A, mRNA expression levels of *SBD*-2 with 10^7^ CFU/mL infection concentration reached maximum, which were also extremely significantly higher than those of the control group (*p* < 0.01) (The extremely significant level was shown in Figure S5, the same below).

Hence, we used 10^7^ CFU/mL infection concentration to optimize the infection time. The result of relative quantification (Figure 1B) showed that mRNA expression levels of *SBD*-2 achieved maximum at 6 h, which were also extremely significantly higher than those of the control group (*p* < 0.01). We also detected SBD-2 protein level using ELISA. As demonstrated in Figure 1C, this standard curve could be used to calculate SBD-2 protein level in samples. Results obviously showed that changes in SBD-2 protein levels shared the same trend with *SBD*-2 mRNA levels that it reached the peak when we used 10^7^ CFU/mL infection concentration (Figure 1D) with 6 h of infection (Figure 1E), which were both extremely significantly higher than those of the control group (*p* < 0.01). Therefore, we took 10^7^ CFU/mL of *E. coli* F17 concentration and 6 h of infection as optimal conditions in subsequent experiments.

### 3.2. Expression Level of SBD-2 Regulates E. coli F17 Resistance in OIECs

**Figure 2:**
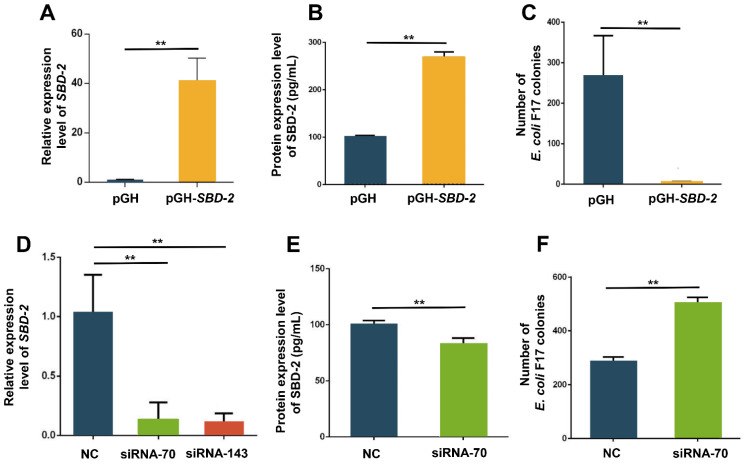
The effect of *SBD*-2 expression on the adhesion of *E. coli* F17 to OIECs. (**A**) Overexpression efficiency of *SBD*-2 mRNA expression level detected by RT-PCR in pGH (Control) cells and pGH-SBD-2 cells. (**B**) Overexpression efficiency of SBD-2 protein expression level detected by ELISA in pGH (Control) cells and pGH-SBD-2 cells. (**C**) Adhesion of the F17 fimbria to OIECs transfected with pGH-SBD-2 compared with pGH (Control) analyzed by bacteria enumeration. (**D**) Interference efficiency of *SBD*-2 mRNA expression level detected by RT-PCR in OIECs transfected with siRNA-SBD-2 compared with negative control. (**E**) Interference efficiency of SBD-2 protein expression level detected by ELISA in OIECs transfected with siRNA-SBD-2 compared with negative control. (**F**) Adhesion of the F17 fimbria to OIECs transfected with siRNA-SBD-2 compared with negative control analyzed by bacteria enumeration. ** *p* < 0.01, extremely significant difference; NS = no difference. Data were shown as mean ± SD, *n* = 3 biological replicates.

To probe into whether *SBD*-2 expression has the function of resisting *E. coli* F17 infection in OIECs, we performed *SBD*-2 overexpression and RNA interference (Figure 2). The results of relative quantification (Figure 2A) and ELISA (Figure 2B) showed mRNA and protein expression levels of SBD-2 in OIECs treated with pGH-SBD-2 were extremely significantly higher than those of pGH treated cells (*p* < 0.01). The result of bacteria enumeration (Figure 2C) showed the remarkably decreased adhesion of *E. coli* F17 to OIECs treated with pGH-SBD-2 compared with that of pGH treated cells (*p* < 0.01). Besides, we used RNA interference to knockdown *SBD*-2 expression. As shown in Figure 2D, the interference efficiency of *SBD*-2 reached more than 50%, and we chose siRNA-70 for next functional verification experiment. The result from ELISA (Figure 2E) showed that SBD-2 protein expression level was significantly reduced in OIECs treated with siRNA-SBD-2 compared with that of non-treated cells (*p* < 0.05). The result from bacteria enumeration (Figure 2F) showed *SBD*-2 knockdown could dramatically improve the adhesion of *E. coli* F17 to OIECs. All these results indicated that overexpression of *SBD*-2 contributed to enhancing *E. coli* F17 resistance in OIECs, while *SBD*-2 knockdown improved adhesion of *E. coli* F17 to OIECs.

### 3.3. Effect of E. coli F17 Stimulation in OIECs on NF-κB and MAPK Pathways

**Figure 3:**
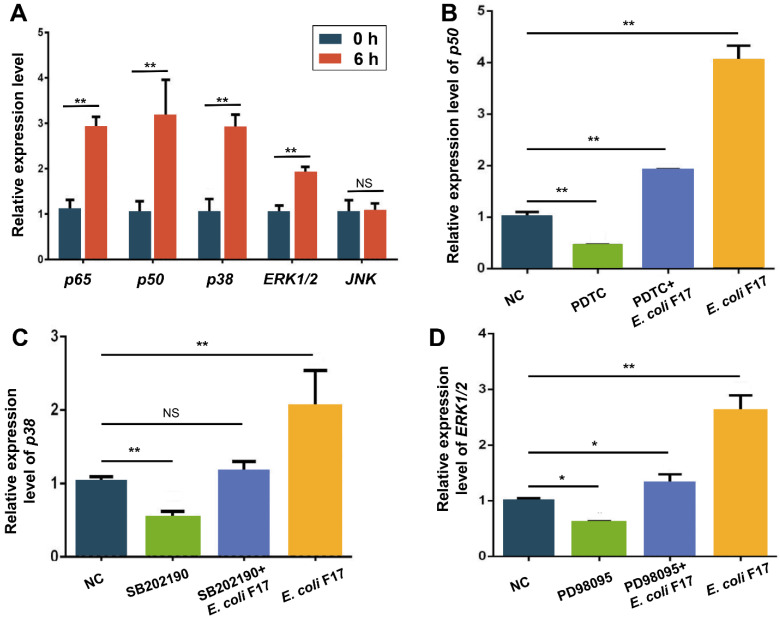
The effect of *E. coli* F17 stimulation in OIECs on NF-κB and MAPK pathways. (**A**) p65, p50, p38, ERK1/2 and JNK expression level determined by RT-PCR in OIECs treated with *E. coli* F17 (10^7^ CFU/mL) for 6 h compared with non-treated control. (**B**) *p50* expression level determined by RT-PCR in OIECs treated with NF-κB inhibitor PDTC, PDTC+*E. coli* F17 and *E. coli* F17 compared with non-treated control. (**C**) *p38* expression level determined by RT-PCR in OIECs treated with p38 MAPK inhibitor SB202190, SB202190+*E. coli* F17 and *E. coli* F17 compared with non-treated control. (**D**) *ERK1/2* expression level determined by RT-PCR in OIECs treated with ERK1/2 MAPK inhibitor PD98095, PD98095+*E. coli* F17 and *E. coli* F17 compared with non-treated control. * *p* < 0.05, significant difference; ** *p* < 0.01, extremely significant difference; NS = no difference. Data were shown as mean ± SD, *n* = 3 biological replicates.

To confirm whether NF-κB and MAPK pathways participating in the process of *E. coli* F17 infection in OIECs, we used 10^7^ CFU/mL *E. coli* F17 to infect OIECs for 6 h. Then, RT-PCR was to detect mRNA expression levels of *p50*, *p65*, *p38*, *ERK1* and *JNK*. The result of relative quantification (Figure 3A) showed that expression levels of *p50*, *p65*, *p38*, *ERK1* and *JNK* were highly up-regulated in infection group compared with non-treated group (*p* < 0.01), which indicated NF-κB and MAPK pathways were activated after *E. coli* F17 stimulating OIECs. Then, we conducted experiments on co-treatment of OIECs with pathway inhibitors and *E. coli* F17. The result of relative quantification (Figure 3B–D) reflected obviously down-regulated mRNA expression levels of *p50* (Figure 3B), *p38* (Figure 3C) and *ERK1/2* (Figure 3D) in inhibitor addition groups in comparison with the non-treated groups (*p* < 0.05 or *p* < 0.01), while mRNA expression levels of *p50* and *ERK1/2* were markedly increased after co-treatment with *E. coli* F17 (*p* < 0.05 or *p* < 0.01). The above results indicated that pathway inhibitors could be used for the subsequent experiments.

### 3.4. NF-κB and MAPK Pathways Influence SBD-2 Expression at the mRNA and Protein Levels

**Figure 4:**
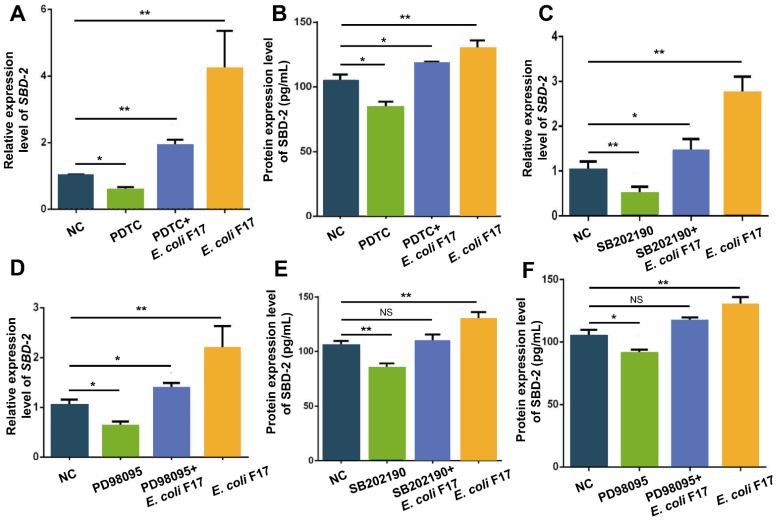
The effect of NF-κB and MAPK pathways on *SBD*-2 expression. (**A**) *SBD*-2 relative expression determined by RT-PCR in OIECs treated with PDTC, PDTC+*E. coli* F17 and *E. coli* F17 compared with non-treated control. (**B**) SBD-2 protein expression determined by ELISA in OIECs treated with NF-κB inhibitor PDTC, PDTC+*E. coli* F17 and *E. coli* F17 compared with non-treated control. (**C**) *SBD*-2 relative expression determined by RT-PCR in OIECs treated with p38 MAPK inhibitor SB202190, SB202190+*E. coli* F17 and *E. coli* F17 compared with non-treated control. (**D**) *SBD*-2 relative expression determined by RT-PCR in OIECs treated with ERK1/2 MAPK inhibitor PD98095, PD98095+*E. coli* F17 and *E. coli* F17 compared with non-treated control. (**E**) SBD-2 protein expression determined by ELISA in OIECs treated with p38 MAPK inhibitor SB202190, SB202190+*E. coli* F17 and *E. coli* F17 compared with non-treated control. (**F**) SBD-2 protein expression determined by ELISA in OIECs treated with ERK1/2 MAPK inhibitor PD98095, PD98095+*E. coli* F17and *E. coli* F17 compared with non-treated control. * *p* < 0.05, significant difference; ** *p* < 0.01, extremely significant difference; NS = no difference. Data were shown as mean ± SD, *n* = 3 biological replicates.

Since we have confirmed that NF-κB and MAPK pathways could be activated after *E. coli* F17 infecting OIECs, the effects of pathways on *SBD*-2 expression is unknown. First, we added NF-κB pathway inhibitor PDTC and *E. coli* F17 infection treatment to OIECs. The relative quantitative results revealed that the mRNA expression level of *SBD*-2 was significantly down-regulated after the addition of PDTC inhibitors compared with non-treated group (*p* < 0.05), then very highly up-regulated after co-treatment with *E. coli* F17 (*p* < 0.01) (Figure 4A). The result of ELISA (Figure 4B) showed the changes of SBD-2 protein level were consistent with the mRNA level. Next, we added p38 pathway inhibitor SB202190 and ERK1/2 pathway inhibitor PD98095, co-treatment with *E. coli* F17. The result of relative quantification (Figure 4C,D) showed an obvious decrease with *SBD*-2 mRNA expression levels in OIECs treated with pathway inhibitors compared with those in non-treated cells (*p* < 0.05 or *p* < 0.01), then highly up-regulated after co-treatment with *E. coli* F17 (*p* < 0.01). The result of ELISA (Figure 4E,F) confirmed that protein level of SBD-2 shared the same trend of mRNA level. Therefore, NF-κB and MAPK pathways could be involved in regulating the *SBD*-2 expression in OIECs infected by *E. coli* F17.

### 3.5. NF-κB and MAPK Pathways Regulate Adhesion of E. coli F17 to OIECs

**Figure 5:**
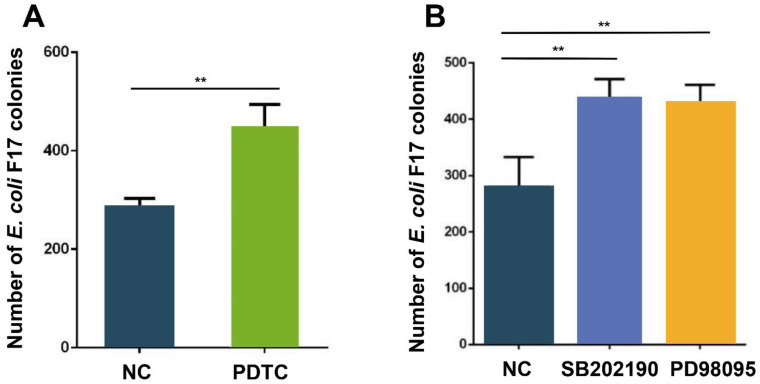
The effect of NF-κB and MAPK pathways on the adhesion of *E. coli* F17 to OIECs. (**A**) Adhesion of the F17 fimbria to OIECs treated with NF-κB inhibitor PDTC compared with negative control analyzed by bacteria enumeration. (**B**) Adhesion of the F17 fimbria to OIECs treated with p38 MAPK inhibitor SB202190 and ERK1/2 MAPK inhibitor PD98095 compared with negative control analyzed by bacteria enumeration. ** *p* < 0.01, extremely significant difference; data were shown as mean ± SD, *n* = 3 biological replicates.

In order to further determine the role of the NF-κB and MAPK pathways in the process of *E. coli* F17 infecting OIECs, NF-κB pathway inhibitor PDTC, p38 pathway inhibitor SB202190 and ERK1/2 pathway inhibitor PD98095 were added to OIECs, respectively, and then collected cells for colony count analysis. The result of bacteria enumeration (Figure 5A) showed that distinctly increased adhesion of *E. coli* F17 to OIECs treated with PDTC compared with that of non-treated cells (*p* < 0.01). Similarly, adhesion of *E. coli* F17 to OIECs were both highly enhanced in OIECs treated with SB202190 and PD98095 contrast with those of non-treated cells (*p* < 0.01) (Figure 5B). These results suggested that NF-κB and MAPK pathways could affect the adhesion of *E. coli* F17 to OIECs.

## 4. Discussion

### 4.1. Effect of SBD-2 Expression on E. coli F17 Adhesion to OIECs

Defensin is a kind of antimicrobial peptide, which exists widely in mammals and has an extensive killing effect on bacteria, fungi, viruses and parasites that invade the body. β-defensin was first found in bovine neutrophils, which is considered to be an important component of the anti-microbial barrier on the mucosal surface [16]. Thus far, *SBD*-1 and *SBD*-2 have been annotated in sheep and are located on chromosome 26. *SBD*-2 is mainly distributed in the epithelial cells of the gastrointestinal tract and digestive tract [17]. In recent years, studies focused on sheep β-defensin seem to be more inclined to study *SBD*-1, because the strong expression of *SBD*-1 in rumen epithelium makes researchers interested in exploring the mechanism of *SBD*-1 expression in the process of sheep rumen innate immunity [18,19,20,21,22]. However, we cannot ignore that sheep colibacillosis is one of the important factors restricting the development of sheep breeding industry in China, which is caused by *E. coli* F17. Previous studies have found that *SBD*-2 expression level is the strongest in the intestinal tract, and the tissue distribution is higher in fetal sheep and newborn lambs, which plays a vital role in developmental regulation before and after birth [23,24]. However, there is no detailed report related to the relationship between *SBD*-2 and *E. coli* F17 infection in OIECs.

In this study, we successfully established a model of *E. coli* F17 infection in OIECs in vitro. To explore the expression changes of *SBD*-2 in OIECs infected by *E. coli* F17, cells were infected with different concentrations of *E. coli* F17. We found that different concentrations of *E. coli* F17 could cause significant changes in *SBD*-2 expression in OIECs, and the expression level of *SBD*-2 in OIECs reached the maximum when the infection concentration was 10^7^ CFU/mL. Further, cells were infected with *E. coli* F17 at the concentration of 10^7^ CFU/mL for different times, and we found that the expression level of *SBD*-2 in OIECs reached the maximum at 6 h. Interestingly, *E. coli* F17 infection markedly upregulated SBD-2 mRNA and protein expression levels in a concentration- and time-dependent manner. Based on the above results, we figured out that the best condition for infection test was *E. coli* F17 infecting OIECs with 10^7^ CFU/mL for 6 h.

Recently, scholars in various fields tend to use gene overexpression technology to explore the mechanism of β-defensin regulating cancer diseases, species including human [25,26], pig [27,28], mice [29,30], etc. Similarly, RNAi technology is also increasingly used in gene functional analysis [31]. However, to comprehensively explore the mechanism of gene action, we are supposed to combine gene overexpression with interference to explore the molecular mechanism of β-defensin [32]. In this study, we carried out *SBD*-2 overexpression and knockdown to explore the ability of *E. coli* F17 adhering to OIECs. The results showed that overexpression of *SBD*-2 could dramatically enhance *E. coli* F17 resistance in OIECs, while interfering with *SBD*-2 could obviously improve the adhesion of *E. coli* F17 to OIECs. Thus, *SBD*-2 is likely to play an essential role in the resistance of OIECs to *E. coli* F17 infection in lambs. It is well established that the distribution of β-defensins differ in different species [33], resulting in their different anti-infection ability to bacteria. In human, Lin et al. reported that Human β-Defensin 118 (*DEFB118*) showed antimicrobial activities against *E. coli* K88 and *E. coli* DH5α with a minimum inhibitory concentration (MIC) of 4 μg/mL [34]. In pig, Su et al. showed that porcine β-defensin 114 (*PBD114*) inhibited the activities of *E. coli* DH5α and K88 with MIC of 64 and 128 μg/mL, respectively [35]. In sheep, we have confirmed the molecular mechanism by which *SBD*-2 regulated the adhesion of *E. coli* F17 to OIECs. Therefore, we believe that *SBD*-2 has antibacterial activity against *E. coli* F17 and has the potential to be used as a novel antibiotic against diarrhea caused by *E. coli* F17 in lambs. It is worth noting that the in vitro cell model cannot completely reflect the subtle changes of the immune environment in vivo. Thus, the function of *SBD*-2 should be further verified in vivo.

### 4.2. Effect of NF-κB and MAPK Pathways on SBD-2 Expression and E. coli F17 Adhesion to OIECs

NF-κB and MAPK signaling pathways are two key pathways activated by *E. coli* induced inflammatory response [36]. The NF-κB family consists of five related transcription factors: p50, p52, REL (also known as cREL), REL-A (p65) and REL-B, which is involved in the differentiation, proliferation and survival of almost all multicellular organisms [37]. The MAPK pathway including ERK1/2, p38 and JNK has been found in mammals. ERK1/2 signaling is mainly involved in the regulation of gene expression, protein translation, cell growth and differentiation. JNK and p38 signaling mainly play a significant role in inflammation, apoptosis and immune system response [38]. A wealth of studies has shown that the expression of induced β-defensin is usually mediated by NF-κB and MAPK pathways [20,39,40,41,42,43]. Among the reports related to Gram-negative bacteria, p38, ERK1/2 and JNK pathways are the most common MAPK pathways that cause inflammatory and immune responses [44,45]. In this study, detected mRNA expression levels of *p50*, *p65*, *p38*, *ERK1/2* and *JNK* were significantly up-regulated after OIECs infected by *E. coli* F17, indicating that MAPK and NF-κB pathways were activated. Furthermore, we added p50 pathway inhibitor PDTC, p38 pathway inhibitor SB202190 and ERK1/2 pathway inhibitor PD98095 to OIECs, respectively. RT-PCR and ELISA analysis showed that the mRNA and protein expression levels of SBD-2 were significantly down-regulated after adding NF-κB and MAPK pathway inhibitors, indicating that *SBD*-2 expression induced by *E. coli* F17 is possibly mediated by NF-κB and MAPK signaling pathways. Finally, in order to more directly clarify the effects of NF-κB and MAPK pathways in OIECs infected by *E. coli* F17, we found that the addition of pathway inhibitors enhanced the adherence of *E. coli* F17 to OIECs. A similar study reported that LPS-induced *SBD*-1 expression is mainly mediated by the TLR4-P38 MAPK pathway [46]. Recently, Su et al. introduced a vital inflammation of NF-κB-dependent that induces porcine β-defensin 114 by using an infected porcine model [47]. Another report showed the novel anti-inflammatory effect of DEFB118 on ETEC-infected mice [48]. Hence, an effective animal model aims to uncover the novel anti-inflammatory function of *SBD*-2 needs to be further excavated, associated with the receptors recognized and the most vital pathway involved in the *E. coli* F17 infection.

## 5. Conclusions

In conclusion, the findings of the present study demonstrate that *E. coli* F17 induces *SBD*-2 expression in OIECs in vitro, which appears to be mediated through the activation of the signaling pathways, NF-κB and MAPK. The regulation of *SBD*-2 expression and the elucidation of the host signaling pathways that contribute to the induction of *SBD*-2 expression are conducive to enhance the innate immune response of the host against bacterial invasion. Our results provide a novel insight for the functionality of *SBD*-2, which could be useful for selecting sheep resisting *E. coli* F17.

## Figures and Tables

**Table 1:** Different treatment of grouping.

A Blank Control Group	B Positive Control Group	C Negative Control Group	D Experimental Group
No treatment	*E. coli* F17 only	NF-κB pathway inhibitor PDTC (25 μM),	NF-κB pathway inhibitor PDTC (25 μM) + *E. coli* F17,
		p38 pathway inhibitor SB202190 (25 μM),	p38 pathway inhibitor SB202190 (25 μM) + *E. coli* F17,
		ERK1/2 pathway inhibitor PD98059 (50 μM),	ERK1/2 pathway inhibitor PD98059 (50 μM) + *E. coli* F17,
		(Add separately)	(Add separately)

## Data Availability

Not applicable.

## Supplementary Materials

The following are available online at https://www.mdpi.com/article/10.3390/biology10121356/s1, Figure S1: Specific isolation method of ovine intestinal epithelial cells, Figure S2: Illustration of pGH-SBD-2 plasmid construction, Figure S3: Ovine intestinal epithelial cells of Hu sheep under inverted microscope, ×40, Figure S4: Agarose gel electrophoresis of cell total RNA, Figure S5: *E. coli* F17 stimulates mRNA and protein expression levels of SBD-2, Table S1: A detailed description of the clinical symptoms, laboratory characteristics and identification of *E. coli* F17, Table S2: Specific primers used for RT-PCR, Table S3: Primers used in plasmid construction, Table S4: Sequence information of RNA oligonucleotides, Table S5: Reaction systems and procedures for PCR.
