# Supplementary material for: Sheep β-Defensin 2 Regulates Escherichia coli F17 Resistance via NF-κB and MAPK Signaling Pathways in Ovine Intestinal Epithelial Cells
Source: Biology (Basel). 2021 Dec 20;10(12):1356. doi: 10.3390/biology10121356 (PMC8698448; doi:10.3390/biology10121356)
Supplement: Supplementary file 1 [file biology-10-01356-s001.zip › biology-1496655-supplementary.pdf]

Supplementary Materials

Table S1. A detailed description of the clinical symptoms, laboratory characteristics and identification of *E. coli* F17.

Clinical symptoms	Identification	Laboratory characteristics
A population including *E. coli* F17-resistant and -sensitive individuals was established based on previous research in our laboratory [1].<br>**a.** We recorded that the number of intestinal bacteria in sensitive group was significantly higher than that in resistant group.<br>**b.** The mucosal tissue in sensitive group was severe damaged, dark and dissolved.<br>**c.** Large lacunae can be seen in the submucosa. The intestinal villi disintegrate and the degree of vascularization is high [2].	We mainly used PCR to identify *E. coli* F17 from lamb faecal samples, referring to Meng et al. [3]<br>**a.** Primers [4]:<br>F-GGGCTGACAGA GGAGGTGGGGC,<br>R-CCCGGCGACAACT TCATCACCGG.<br>**b.** Reaction system: 1×T3 Super PCR Mix (Tsingke) 19 μL, forward primer 1 μL, reverse primer 1μL, bacterial DNA 4 μL.<br>**c.** Reaction procedure: 98°C for 30 s, 58°C for 10 s, 72°C for 5 s, followed by 34 cycles.<br>**d.** The PCR product was identified by 1.5% agarose gel electrophoresis, and the target fragment should be 411 bp [4].	In this study, when we treated ovine small intestinal epithelial cells with *E. coli* F17 bacteria liquid, we observed that with the increase of *E. coli* F17 bacterial concentration, the cell growth condition went from bad to worse, mainly reflected in the increased number of dead cells and the weakening of adherent growth ability, which is compared with non-treated cells.

Table S2. Specific primers used for RT-PCR.

Name	Primer Name	Sequence (5′-3′)	GenBank accession	Product Length (bp)	Annealing temperature (°C)
SBD2	*SBD2*-F	CTCGTGCTCTTCTTCGTGGT	NM_001198545.1	90	60
	SBD2-R	GATGCCTTTCTTCCAACGGC			
p50	*P50*-F	GAAGTGCAAAGGAAACGACA	XM_042251202.1	156	58
	P50-R	ATAGTGGGGGAAGCCATACC			
P65	*P65*-F	GGATGGCTTCTATGAGGCTG	XM_027959295.2	135	58
	P65-R	AAGGGGTTGTTGTTGGTCTG			
P38	*P38*-F	CTTGGATTTTGGGTTGGCAC	NM_001142894.1	184	58
	P38-R	GTCTTTCCCCTTGAACAGCG			
ERK1/2	*ERK1/2*-F	CTGTTCCCGAATGCTGACTC	XM_012157699.1	135	58
	ERK1/2-R	CTCGTCGCTTGGATCGTAGT			
JNK	*JNK*-F	ATTTATGGTCTGTGGGGTGC	XM_004002020.3	115	58
	JNK-R	CATGGTGTTCCAAGCTGTTC			
GAPDH	*GAPDH*-F	TCTCAAGGGCATTCTAGGCTAC	NM_001190390.1	151	60
	GAPDH-R	GCCGAATTCATTGTCGTACCAG			

Table S3. Primers used in plasmid construction

Name	Primer Name	Sequence (5’-3’)	Product length (bp)	Annealing temperature (°C)
SBD2	*SBD2*-F	GATATCATGAGGCTCCATCACCTGCT CCTCGTGCTCTTCTTCGTGGT	195	60
	SBD2-R	GATATCTTACTTCAGTCTGCAGCATT TTACTGGGGGCCCGAAACAGGT		

Note. the underlined nucleotides indicate restriction enzyme sites.

Table S4. Sequence information of RNA oligonucleotides.

Name	Sequence Name	Sequence (5′-3′)
SBD2	siRNA-70	sense: GGAGUAACAGAUAGUCUAATT
		antisense: UUAGACUAUCUGUUACUCCTT
	siRNA-143	sense: GACAGAUUGGCACCUGUUUTT
		antisense: AAACAGGUGCCAAUCUGUCTT
	Negative control	sense: UUCUCCGAACGUGUCACGUTT
		antisense: ACGUGACACGUUCGGAGAATT

Table S5. Reaction systems and procedures for PCR.

**Reverse transcription**	**RT-PCR**
**a.** Reaction system	**a.** Reaction system
5×FastKing-RT Super Mix 4 μL,	2×TSINGKE Master qPCR Mix (SYBR Green I) 10 μL,
total RNA 1000 ng,	Forward primer 0.8 μL (10 μM),
RNase-free ddH2O up to 20 μL.	Reverse primer 0.8 μL (10 μM),
**b.** Reaction procedure	ddH_2O up to 20 μL.
42°C for 15 min,	**b.** Reaction procedure
95°C for 3 min,	95°C for 30 s,
4°C forever.	40 cycles of 95°C for 10 s, 60°C for 30 s.

Isolation of small intestinal epithelial cells from Hu Sheep by Enzymatic digestion

↓

a. Small intestine tissue was immersed in PBS containing 5% penicillin-streptomycin, meanwhile, remove the mesentery and intestinal contents.

↓

b. The intestinal canal was cut into small pieces around 1mm^3 sharp scissors. The fragments were transferred into a 15 mL centrifuge tube, wash four times with DMEM/F12 medium.

↓

c. Then incubated at 37°C shake digestion for 30 min in the presence of type IV collagenase (300U/ml).

↓

d. Add the same volume of DMEM/F12 medium to stop digestion, centrifuge at 1000 rpm for 5 min, then discard the supernatant. Wash twice with PBS.

↓

e. Primary culture at 37 °C in 5% CO^2 incubator. According to the sensitivity of different cells to trypsin, small intestinal epithelial cells were purified by different time of trypsin digestion.

Figure S1. Specific isolation method of ovine intestinal epithelial cells.

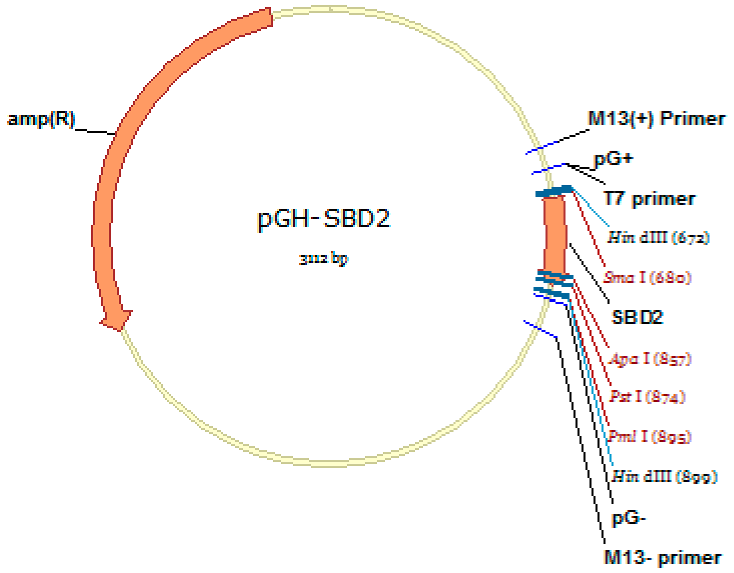

Figure S2. Illustration of pGH-SBD-2 plasmid construction.

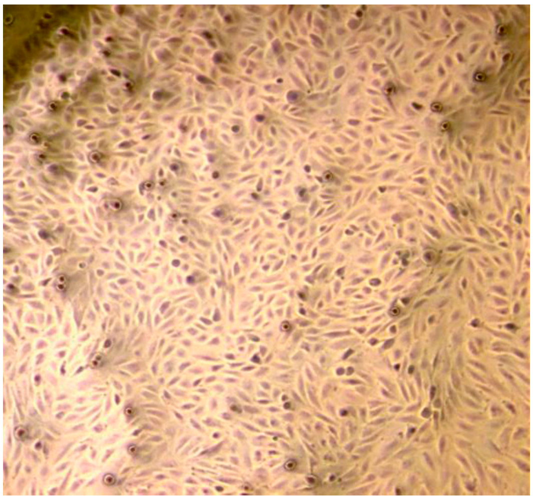

Figure S3. Ovine intestinal epithelial cells of Hu sheep under inverted microscope, ×40.

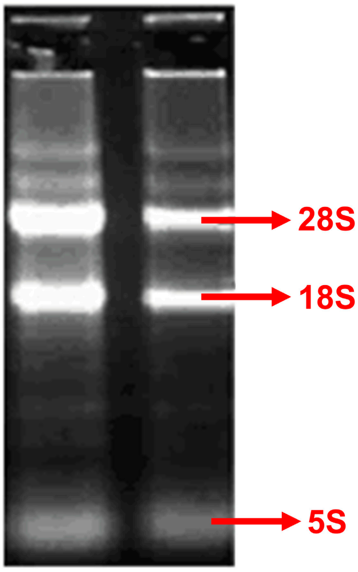

Figure S4. Agarose gel electrophoresis of cell total RNA.

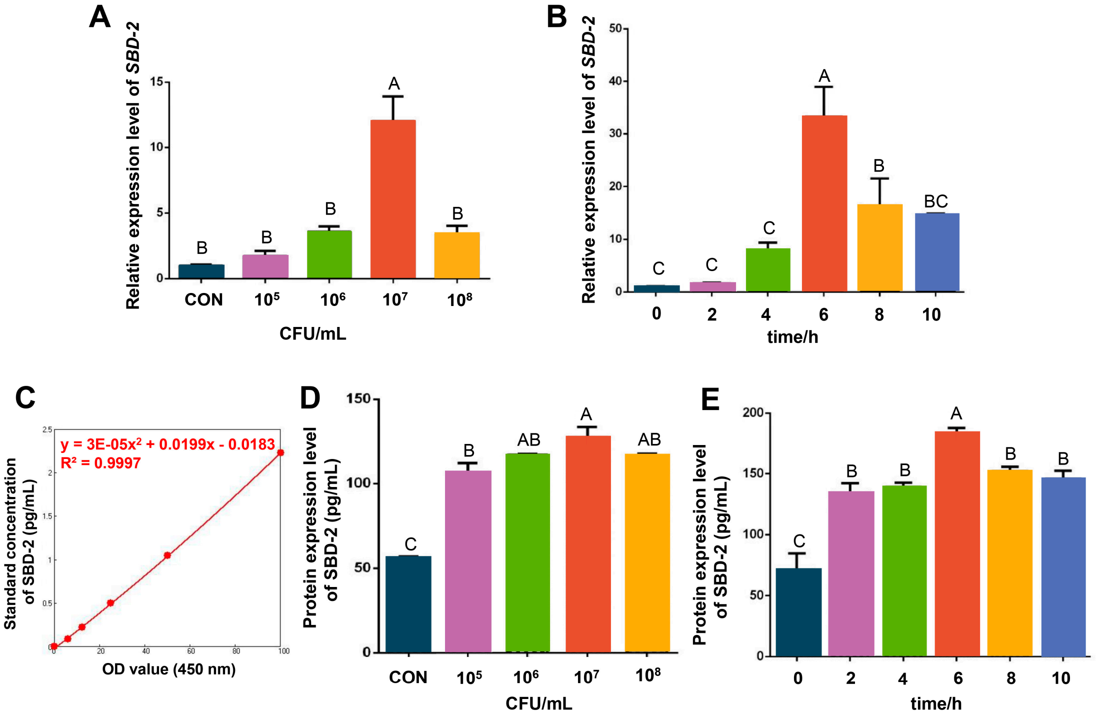

Figure S5. *E. coli* F17 stimulates mRNA and protein expression levels of SBD-2. **A** *SBD-2* mRNA expression levels detected by RT-PCR in OIECs treated with the indicated concentrations of *E. coli* F17 compared with non-treated controls. **B** *SBD-2* mRNA expression levels detected by RT-PCR in OIECs treated with *E. coli* F17 (10^7 CFU/mL) for various time intervals compared with non-treated controls. **C** Standard curve drawn by SBD-2 protein concentration value (y axis) and OD value at 450 nm (x axis). **D** SBD-2 protein expression levels detected by ELISA in OIECs treated with the indicated concentrations of *E. coli* F17 compared with untreated controls. **E** SBD-2 protein expression levels detected by ELISA in OIECs treated with *E. coli* F17 (10^7 CFU/mL) for various time intervals compared with untreated controls. Mean values with different letters in the same row are significantly different ($p < 0.05$) according to Duncan's multiple range test; data were shown as mean ± SD, n = 3 biological replicates.

Reference

1. Jin, C.; Bao, J.; Wang, Y.; Chen, W.; Zou, S.; Wu, T.; Wang, L.; Lv, X.; Gao, W.; Wang, B., et al. Changes in circRNA expression profiles related to the antagonistic effects of Escherichia coli F17 in lamb spleens. *Scientific reports* **2018**, *8*, 14524, doi:10.1038/s41598-018-31719-5.
2. Jin, C.; Bao, J.; Wang, Y.; Chen, W.; Wu, T.; Wang, L.; Lv, X.; Gao, W.; Wang, B.; Zhu, G., et al. Changes in long non-coding RNA expression profiles related to the antagonistic effects of Escherichia coli F17 on lamb spleens. *Scientific reports* **2018**, *8*, 16514, doi:10.1038/s41598-018-34291-0.
3. Liu, W.; Yuan, C.; Meng, X.; Du, Y.; Gao, R.; Tang, J.; Shi, D. Frequency of virulence factors in Escherichia coli isolated from suckling pigs with diarrhoea in China. *Veterinary journal (London, England : 1997)* **2014**, *199*, 286-289, doi:10.1016/j.tvjl.2013.11.019.
4. Vu Khac, H.; Holoda, E.; Pilipcinec, E.; Blanco, M.; Blanco, J.E.; Mora, A.; Dahbi, G.; López, C.; González, E.A.; Blanco, J. Serotypes, virulence genes, and PFGE profiles of Escherichia coli isolated from pigs with postweaning diarrhoea in Slovakia. *BMC veterinary research* **2006**, *2*, 10, doi:10.1186/1746-6148-2-10.
